# Correction to “Successful Haploidentical Hematopoietic Stem Cell Transplantation for Paroxysmal Nocturnal Hemoglobinuria With Severe Pancytopenia Developed After Long‐Term Aplastic Anemia Treatment”

**DOI:** 10.1002/ccr3.70838

**Published:** 2025-09-04

Sakurai K Saito K Hatta S Katsuoka Y Meguro K Yokoyama H Izumi T Successful haploidentical hematopoietic stem cell transplantation for paroxysmal nocturnal hemoglobinuria with severe pancytopenia developed after long‐term aplastic anemia treatment Clinical Case Reports 2024 12:e8832 10.1002/ccr3.8832.38681032 PMC11053247

In the sentence “the conditioning regimen…” under the case presentation section (page 2, right column, line 5), the text “cyclophosphamide (14.5 mg/m^2^ for 2 days)” was incorrect. This should read as “cyclophosphamide (14.5 mg/kg for 2 days).”

We apologize for this error.

## Author Contributions

**Kazuki Sakurai:** data curation, investigation, methodology, writing – original draft, writing – review and editing. **Kei Saito:** writing – review and editing. **Yuna Katsuoka:** data curation, writing – review and editing. **Kuniaki Meguro:** data curation, writing – review and editing. **Hisayuki Yokoyama:** conceptualization, writing – review and editing. **Toru Izumi:** project administration.

